# Pentraxin-3, procalcitonin and lactate as prognostic markers in patients with sepsis and septic shock

**DOI:** 10.18632/oncotarget.23701

**Published:** 2017-12-26

**Authors:** Chenggong Hu, Yongfang Zhou, Chang Liu, Yan Kang

**Affiliations:** ^1^ Department of Critical Care Medicine, West China Hospital of Sichuan University, Sichuan 610041, Nanchong, China

**Keywords:** sepsis, lactate, mortality, pentraxin-3, procalcitonin

## Abstract

The purpose of this study was to confirm the prognostic value of pentraxin-3 (PTX3), procalcitonin (PCT) and lactate in patients with severe infections requiring ICU management and to develop and validate a model to enhance mortality prediction by combining severity scores with biomarkers. We included 141 patients with the diagnosis of sepsis/septic shock. The levels of PTX3, PCT and lactate were measured on day 0, 3, 7 of hospitalization and Sequential Organ Failure Assessment (SOFA) and Acute Physiology and Chronic Health Evaluation II (APACHE II) scores were also evaluated. The influence of these variables on 28-day mortality was evaluated. The 28-day mortality rate in this study was 28.8%. The baseline levels of PTX3, PCT and lactate in the non-survival group were higher than in the survival group (P < 0.05 for all). Pearson's correlation found that PTX3, PCT and lactate were all positively correlated with SOFA and APACHE II scores (P <0.01 for all). Univariate and multivariate Cox regression revealed that PTX3, PCT and lactate were independently associated with 28-day mortality. The models combining above three biomarkers performed better predictive property than each individual one as determined by receiver operating characteristic (ROC) analysis. In summary, our results suggest that PTX3, PCT and lactate could serve as clinically informative biomarkers of disease severity and patient outcome in sepsis/septic shock. A model combining PTX3, PCT and lactate improves mortality prediction in these patients.

## INTRODUCTION

Sepsis is a major cause of intensive care unit (ICU) admission [1]. Although mortality has decreased in the past decade due to increased awareness and improved management, the short-term mortality remains 20% or more [2, 3]. Early stratification and recognition of patients at higher risk of death pose serious clinical challenges, since timely decisions on the best therapeutic approach and appropriate site of care are both crucial in this healthcare setting [4]. Thus, it is urgent to find effective factors for the prognosis of sepsis at the early stage.

Concurrently, various biomarkers have been proposed. The most widely studied biomarker in patients with sepsis or septic shock is procalcitonin (PCT). Although PCT is associated with the severity of systemic infection and the presence of organ dysfunction and dynamic changes of PCT could be predictive of certain outcomes [5, 6], In addition, emerging interest focus on the role of lactate as a biomarker of risk in the critically ill patients [7]. Lactate has been widely used as a marker of altered tissue perfusion. Increases in the concentrations are associated with higher mortality rates [8] and lactate clearance during hospitalization is a predictor for decreased mortality [9]. However, their limited performance precludes using these markers for individual risk stratification and personalization of decision-making processes [10, 11]. Pentraxin 3 (PTX3) is an acute phase protein representing the long pentraxin subfamily [12] and is expressed in various cells, like monocytes, endothelial cells, dendritic cells or neutrophils during inflammatory processes [13]. As a multifunctional pattern-recognition molecule, it has been reported to be strongly associated with the severity of infection [14]. PTX3, PCT, and lactate are all detectable in patients with sepsis within a large time window after the onset of sepsis, while other novel pro-inflammatory cytokines (such as interleukin and tumor necrosis factor-a) have a short window of expression, even they showed good prognostic values for mortality.

In our study, a prospective analysis was conducted to confirm the prognostic value of PTX3. We then investigate the dynamic change of PTX3, PCT and lactate in patients with sepsis during the first week in ICU stay and perform a comparison with well-established scores, such as Sepsis-related Organ Failure Assessment (SOFA) and Acute Physiology and Chronic Health Evaluation (APACHE II). We also hypothesized that the mortality prediction by using the combination of the above three parameters would significant improve in patients with sepsis/septic shock.

## RESULTS

### Patient demographics

After exclusion, 141 patients were administrated in the study, including 86 males and 55 females. During the follow up period, 42 patients were dead, accounting for 28.8% of all the subjects. Baseline features that associated with 28-day mortality based on the univariate analysis were pre-existing hypertension and chronic renal failure, occurrence of septic shock and acute kidney injury, SOFA score and APACHE II score at admission (Table 1). The most common areas of infection were lungs (46, 32.6%) followed by abdomen/pelvis (34, 24.1%), urinary system (22, 15.6%) and blood (18, 12.8%), but no significance difference in area infected between two groups were observed (P=0.159). The percentage of patients with septic shock and acute kidney injury was significantly higher in the non-survival group than those in the survival group. With respective to laboratory results, patients in the non-survival groups had higher levels of white blood cell, CRP, PCT, creatinine, lactate, lactate clearance (LCR), NT-proBNP, glucose, total bilirubin, PTX3 and lower level of albumin, compared with those in the survival group. No significant differences in patients’ age, gender, BMI, ICU days and other laboratory values were found between the survival and non-survival groups.

**Table 1:** Baseline characteristics of the study subjects, according to outcome of 28-days mortality

Clinical characteristics	Total (n=141)	Survivors (n=99)	Non-survivors (n=42)	P
Age (years)	64 (33-78)	61 (33-71)	69 (42-78)	0.135
Gender (male, %)	86 (61)	58 (59)	28 (67)	0.477
BMI kg/m^2^	26.5±4.6	26.2±3.6	27.1±4.4	0.207
ICU days	13 (2-22)	13 (3-19)	11 (2-22)	0.439
Comorbidities				
Hypertension	60 (42.6)	36 (36.4)	24 (57.1)	0.036
Chronic coronary disease	28 (19.6)	18 (18.2)	10 (23.8)	0.593
Diabetes mellitus	38 (27.0)	22 (22.2)	16 (38.1)	0.083
Chronic renal failure	32 (22.7)	16 (16.2)	16 (38.1)	0.009
Chronic liver disease,	10 (7.1)	6 (6.1)	4 (9.5)	0.484
COPD	14 (9.9)	9 (9.1)	5 (11.9)	0.759
Site of infection (%)				0.159
Respiratory	46 (32.6)	32 (32.3)	14 (33.3)	
Abdominal/pelvic	34 (24.1)	27 (27.3)	7 (16.7)	
Urinary	22 (15.6)	17 (17.2)	5 (11.9)	
Blood	18 (12.8)	8 (8.1)	10 (23.9)	
Skin and soft tissue	12 (8.5)	8 (8.1)	4 (9.5)	
Others	9 (6.4)	7 (7.0)	2 (4.7)	
Patient evaluation				
Septic shock (%)	37 (26.2)	17 (17.2)	20 (47.6)	0.004
Acute kidney injury (%)	22 (15.6)	10 (10.1)	12 (28.6)	0.012
Acute liver injury (%)	21 (14.9)	12 (12.1)	9 (21.4)	0.246
SOFA score	5 (2-14)	5 (2-8)	7 (3-14)	<0.001
APACHE II score	21 (10-36)	20 (10-27)	25 (16-36)	<0.001
Laboratorial results				
Hemoglobin (g/dL)	9.4±2.3	9.7±2.6	9.2±2.0	0.267
white blood cell (×10^9^/L)	15.6(8.9-27.7)	14.3(8.9-18.3)	17.6(10.5-27.7)	0.017
CRP (mg/dl)	91(56.3-285)	88.2(56.3-206)	105(99.8-285)	0.009
Procalcitonin (ng/ml)^*^	2.8(0.13-38.7)	2.6(0.13-25.9)	5.5(0.9-38.7)	<0.001
BUN (mg/dL)^*^	13.9(1.9-79.0)	13.1(3.7-45.5)	16.7(1.9-79.0)	0.176
Creatinine (mg/dL)	114±19.9	107±17.6	120±20.2	0.013
Lactate (mmol/L)^*^	1.81 (1.18-3.27)	1.74 (1.18-2.30)	1.97 (1.46-3.27)	<0.001
24 h LCR (%)	32.2±6.0	39.3±5.2	20.9±5.4	<0.001
INR^*^	1.2 (1.1-2.0)	1.2 (1.1-1.4)	1.4 (1.2-2.0)	0.453
Albumin (g/L)^*^	28.7±6.2	30.2±7.5	26.9±6.1	0.013
NT-proBNP (pg/ml)^*^	4102(499-11587)	1945(499-9536)	6525(973-11587)	0.001
Glucose (mmol/L)	11.9±1.9	10.5±3.4	14.4±3.2	0.027
Total bilirubin (μmol/L)	29.9±6.9	28.3±6.1	32.1 ± 5.5	0.007
PTX3 (ng/mL)	50.7 (25.4-111.1)	45.2 (25.4-86.1)	65.7 (30.9-111.1)	<0.001

Note: BMI= body mass index; COPD=chronic obstructive pulmonary disease; CRP=C-reactive protein; BUN= blood urea nitrogen; INR=international normalized ratio; LCR= lactate clearance; NT-proBNP=N-terminal prohormone of brain natriuretic peptide; PSP= pancreatic stone protein. ^*^ variables with non-normal distribution and demonstrated as median (range).

### Correlation of PTX3, PCT, and lactate with SOFA and APACHE score

We analyzed the relationship among APACHE II and SOFA scores and laboratory results of the overall group. Interesting, we identified several biomarkers that corrected with SOFA or APACHE II scores (Table 2). Among this, PTX3 showed most close correlation with SOFA score (r=0.594, P<0.001) and APACHE II score (r=0.411, P<0.001), flowed by PCT, which was positively correlated with SOFA score (r=0.531, P<0.001) and APACHE II score (r=0.390, P<0.001). Lactate, 24 h LCR, CRP and white blood cell were also positively correlated with two scores. Besides, albumin showed a distinctly negative correlation with SOFA score (r=-0.329, P<0.001), but not APACHE II score (r=-0.189, P=0.079).

**Table 2:** Correlations among SOFA and APACHE II scores and serum concentrations of studied biomarkers in the overall group of patients

Parameters	SOFA score		APACHE II score	
	r	P values	r	P values
Hemoglobin (g/dL)	0.117	0.113	0.030	0.349
white blood cell (×10^9^/L)	0.284	0.011	0.217	0.029
CRP (mg/dl)	0.309	0.001	0.304	0.003
Procalcitonin (ng/ml)	0.531	<0.001	0.390	<0.001
BUN (mg/dL)	0.093	0.365	0.137	0.083
Creatinine (mg/dL)	0.104	0.181	0.087	0.061
Lactate (mg/dl)	0.462	<0.001	0.336	<0.001
INR	0.155	0.166	0.074	0.283
Albumin (g/L)	-0.329	<0.001	-0.189	0.079
NT-proBNP (pg/ml)	0.278	0.013	0.139	0.080
Glucose (mmol/L)	0.194	0.038	0.113	0.117
Total bilirubin (μmol/L)	0.132	0.094	0.017	0.520
PTX3	0.594	<0.001	0.411	<0.001

Note: r= correlation coefficient; CRP=C-reactive protein; BUN= blood urea nitrogen; INR=international normalized ratio; LCR= lactate clearance; NT-proBNP=N-terminal prohormone of brain natriuretic peptide.

### Changes of SOFA, APACHE score and studied biomarkers

**Figure 1:**
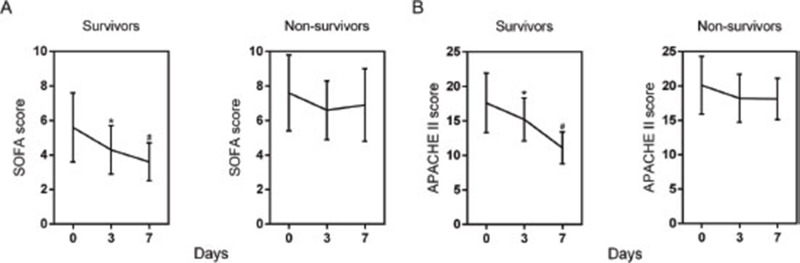
Changes of SOFA **(A)** and APACHE II **(B)** scores of patients with sepsis during the first 7 days of ICU stay. Data are shown as mean (95% confidence interval). ^*^ P<0.01 compared with day 0; ^#^ P<0.01 compared with day 3.

**Figure 2:**
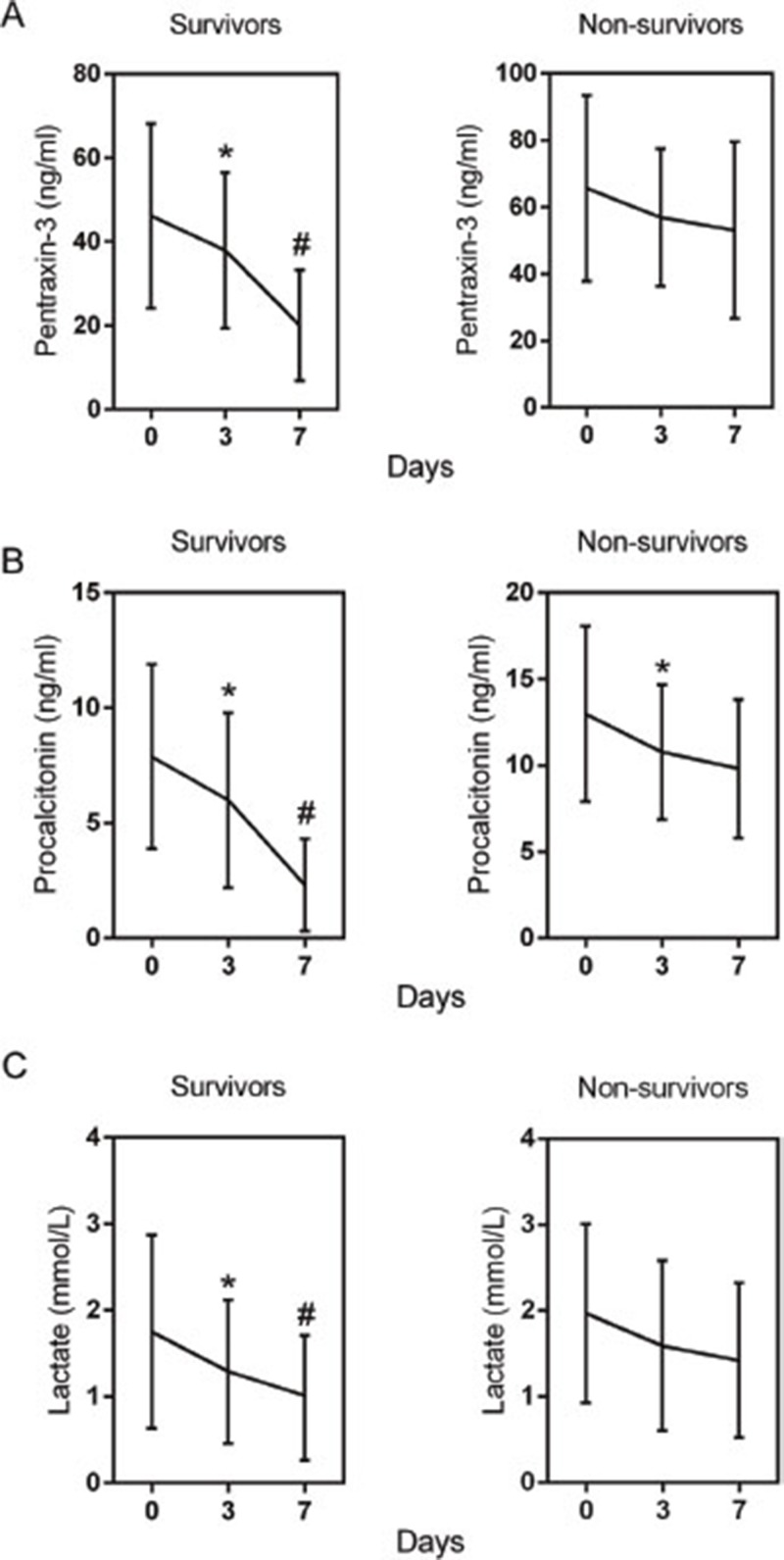
Changes of serum concentrations of pentraxin-3 **(A)**, procalcitonin **(B)**, and lactate **(C)** of patients with severe sepsis during the first 7 days of ICU stay. Data are shown as mean (95% confidence interval). ^*^ P<0.01 compared with day 0; ^#^ P<0.01 compared with day 3.

We then analyzed the changes of SOFA, APACHE score and studied biomarkers. As shown in Figure 1, a significant decrease trend in the SOFA and APACHE score were observed in survival group during the first 7 days, but no significant change of them in non-survival group. Besides, the changes were also compared among three biomarkers. As expected, all of PTX3 (Figure 2A), PCT (Figure 2B) and lactate (Figure 2C) showed remarkable reduction at day 3 from day 0 and further reduced at day 7 from day 3 in survival group. Only PCT showed significant decrease at day 3 from day 0 in ICU stay in non-survival group (Figure 2B). Nevertheless, when the differences in the dynamic change of biomarker levels between the two groups were compared, only the differences in PTX3 and PCT concentrations from day 0 to day 7 achieved statistical significance (Table 3).

**Table 3:** Changes in study biomarkers during the first week of ICU stay in patients with sepsis

Biomarker	Survivors	Non-survivors	P values
PTX3 (ng/ml) (% change)			
Day 0 to 3	17 (4; 31)	6 (-4; 13)	<0.001
Day 0 to 7	38 (14; 117)	10 (-7; 20)	<0.001
Procalcitonin (ng/ml) (% change)			
Day 0 to 3	14 (4; 39)	9 (-3; 28)	0.092
Day 0 to 7	56 (22; 165)	14 (-10; 57)	<0.001
Lactate (mg/dl) (% change)			
Day 0 to 3	10 (3; 20)	4 (-6; 14)	0.232
Day 0 to 7	24 (10; 42)	13 (8; 31)	0.078

Note: 1. The change of the study markers were expressed as percentages of baseline concentrations at day 0 and were obtained according the following formula: (day 0 – day 2 or 5)/day 0 × 100.

### Predictive value of PTX3, PCT and lactate

As illustrated in Table 4, univariate Cox regression analysis revealed that septic shock, SOFA score, APACHE II score, PCT, lactate and glucose were all related with 28-day mortality in patients with sepsis. Multivariate analysis was then performed in order to delineate various prognostic indicators. After adjusting for other risk factors, SOFA score was an independent risk factor for 28-day mortality (HR=1.78; 95%CI: 1.25–2.34; P<0.001), but not APACHE II score (HR = 1.16; 95% CI: 0.97–1.45; P=0.134). Additionally, PCT (HR = 2.41; 95% CI: 1.38–4.31; P<0.001), lactate (HR = 1.55; 95% CI: 1.06–2.29 P=0.134), and PTX3 (HR = 2.21; 95% CI: 1.19–5.15; P<0.001) remained independently related with 28-day mortality.

**Table 4:** Univariate and multiple Cox proportional hazards analysis of 28-day mortality

Variables	Univariate		Multivariate	
	HR (95%CI)	P values	HR (95%CI)	P values
Age (years)	0.74 (0.37–1.48)	0.399	NI	
BMI kg/m2	0.71 (0.43–1.15)	0.172	NI	
ICU days	1.27 (0.98–1.64)	0.068	NI	
Septic shock	1.50 (1.20–1.85)	<0.001	1.39 (1.10–1.76)	0.006
Acute kidney injury	1.17 (0.85–1.66)	0.254	NI	
SOFA score	1.95 (1.28–2.42)	<0.001	1.78 (1.25–2.34)	<0.001
APACHE II score	1.29 (1.02–1.71)	0.035	1.16 (0.97–1.45)	0.134
white blood cell (×109/L)	1.21 (0.85–1.72)	0.279	NI	
CRP (mg/dl)	1.38 (0.92–1.96)	0.097		
Procalcitonin (ng/ml)	2.54 (1.45–4.44)	<0.001	2.41 (1.38–4.31)	<0.001
Creatinine (mg/dL)	1.13 (0.80–1.61)	0.403	NI	
Lactate (mg/dl)	1.61 (1.14–2.43)	<0.001	1.55 (1.06–2.29)	0.001
INR	0.92 (0.72–1.66)	0.449	NI	
Albumin (g/L)	0.94 (0.77–1.10)	0.215	NI	
NT-proBNP (pg/ml)	1.34 (0.67–2.91)	0.309	NI	
Glucose (mmol/L)	1.07 (0.52–1.62)	0.535	NI	
PTX3 (mmol/L)	2.36 (1.25–5.46)	<0.001	2.21 (1.19–5.15)	<0.001
Total bilirubin (μmol/L)	0.89 (0.51–1.64)	0.535	NI	

Note: BMI= body mass index; CRP=C-reactive protein; LCR= lactate clearance; NT-proBNP=N-terminal prohormone of brain natriuretic peptide; CI= confidence interval; HR= odds ratio; NI= not included in multivariate survival analysis.

### Identification of the optimal cut-off value

**Figure 3:**
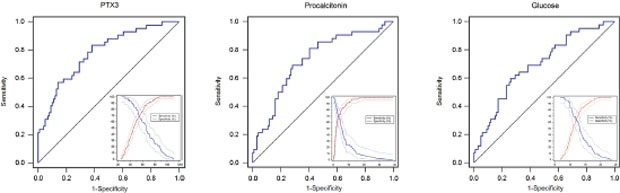
Receiver operating characteristics (ROC) curves and plot versus criterion value curves in the lower right part were used to discriminate survivors compared to non-survivors with sepsis based on baseline pentraxin-3 **(A)**, procalcitonin **(B)**, and lactate **(C)**. The X-axis shows the level of study markers and the Y-axis shows the percentage. The solid and dashed lines indicate sensitivity and specificity with 95% confidence intervals, respectively.

**Figure 4:**
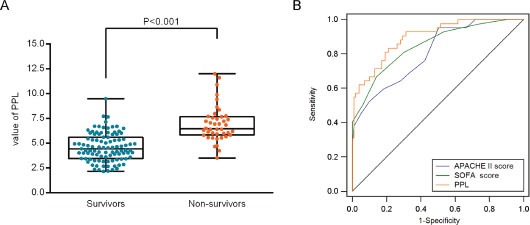
Prognostic value of the new risk factor named PPL (on the basis of pentraxin-3, PCT, and lactate) in 28-day mortality **(A)** The value of PPL in the survival and non-survival group. **(B)** Comparison of receiver operating characteristic (ROC) analysis of SOFA score, APACHE II score and PPL in 28-day mortality of patients with sepsis.

**Figure 5:**
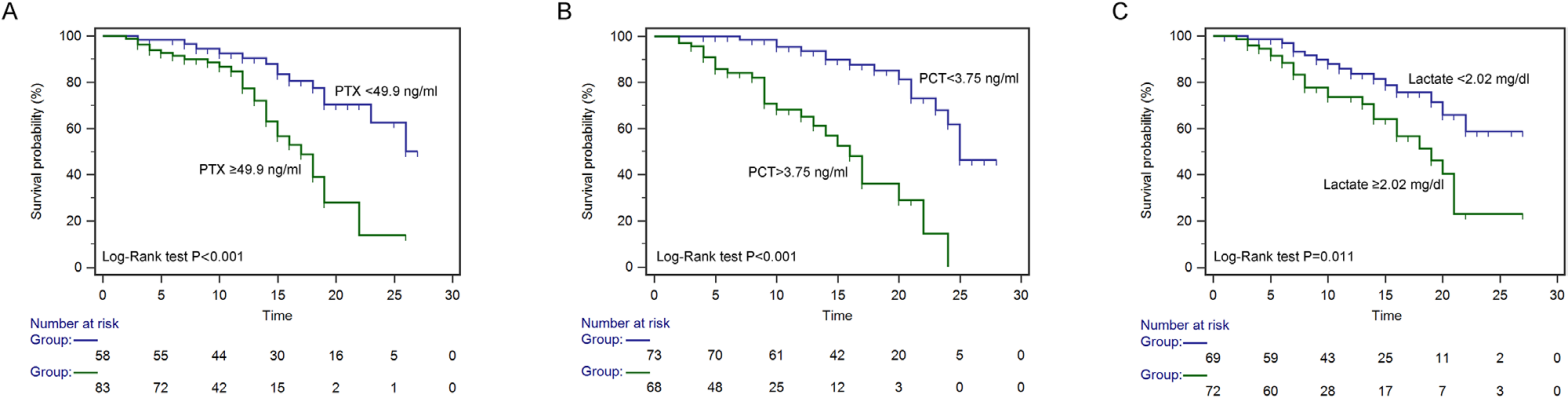
Kaplan-Meier curves of 28-day mortality in patients with sepsis stratified by the cut-off value of pentraxin-3 **(A)**, PCT **(B)** and lactate **(C)**.

**Figure 6:**
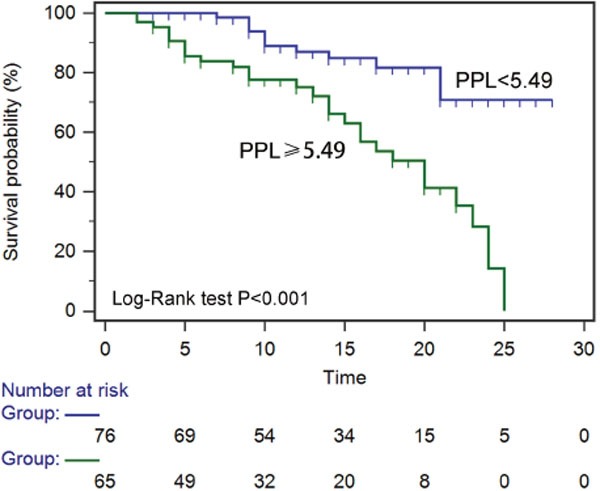
Kaplan-Meier curves of 28-day mortality in patients with sepsis stratified by the value of PPL (pentraxin-3+procalcitonin+lactate)

Receiver operating characteristic (ROC) curves were then used to determine the optimal cut-off value for predicting 28-day mortality. The results showed that area under the curve (AUC) values of PTX3, PCT and lactate were 0.73 (95%CI: 0.65-0.81), 0.66 (95%CI: 0.57-0.74) and 0.78 (95%CI: 0.70-0.84), respectively. The optimal cut-off was 49.9 ng/ml for PTX3 (Figure 3A), 3.75 ng/ml for PCT (Figure 3B) and 2.02 mg/dl for lactate (Figure 3C) at baseline. The sensitivity and specificity of each cutoff values were shown in Table 4. Furthermore, SPFA score (AUC:0.85, 95%CI: 0.77-0.88) and APACHE II score (AUC:0.81, 95%CI: 0.73-0.87) seemed to show more satisfied prognostic value for 28-day mortality (Table 5). Subsequently, above three independent factors identified through multivariate analysis were combined to generate a new prognostic score, which we named the PPL. This was calculated as follows: 0.065^*^PTX3 + 0.152^*^PCT + 0.142^*^lactate. The value of PPL showed dramatically increased in the non-survival group compared to the survival group (Figure 4A). Accordingly, the ROC curve comparison showed a superior prognostic property of PPL (AUC:0.90, 95%CI: 0.83-0.94) compared with SPFA score and APACHE II score (P <0.001, Table 5 and Figure 4B). Patients were assigned into two groups according to each cut-off value achieved above and survival rates were then analyzed through Kaplan–Meier analysis and log-rank test. Figure 5A revealed higher mortality of the patient group with PTX>49.9 ng/ml (P<0.001). Similar results were observed in patient group with PCT> 3.75 ng/ml (Figure 5B, P<0.001) or lactate>2.02 mg/dl (Figure 5C, P=0.011). Consistent with the above results, PPL demonstrated the highest HR, thus showing the best discriminatory ability (Figure 6) (HR = 2.75, 95% CI:1.46–4.11, P <0.001).

**Table 5:** The receiver operating characteristic (ROC) analysis of SPFA score, APACHE II score and three laboratory values for prediction prognosis

Biomarkers	AUC (95%CI)	P value	Youden	Cut-off	Sensitivity	Specificity
Procalcitonin (ng/ml)	0.73(0.65-0.81)	<0.001	0.50	3.75	71.1%	72.7%
Lactate (mg/dl)	0.67(0.59-0.75)	0.001	0.35	2.02	42.8%	91.9%
PTX3 (ng/ml)	0.78(0.70-0.84)	<0.001	0.46	49.9	83.3%	64.2%
SPFA score	0.85(0.77-0.88)	<0.001	0.64	0.42	76.0%	93.1%
APACHE II score	0.81(0.73-0.87)	<0.001	0.54	17	92.3%	51.0%
PPL	0.90(0.83-0.94)	<0.001	0.69	5.49	90.6%	83.8%

Note: AUC, area under the receiver operating characteristic curve; SE, standard error; CI, confidence interval; PPL= 0.065^*^PTX3 + 0.152^*^PCT + 0.142^*^lactate. P-value, compared with AUC of 0.5.

## DISCUSSION

The study evaluated the accuracy of PTX3, PCT and lactate, and their combinations, as biomarkers of severity and prognosis of patients with sepsis/septic shock. Using APACHE II and SOFA scores as the reference of the severity of sepsis [30, 31], a significant correlation was identified between the two scores and the concentrations of PTX3, PCT and lactate. Elevated PTX3, PCT and lactate at baseline were all closely associated with poor outcome and were risk factors for 28-day morality. We demonstrated that PTX3 had comparable prognostic value with PCT and superior to lactate for the prediction of 28-day mortality. Moreover, we developed a sepsis model, based on above three biomarkers, to predict in-hospital mortality in patients with sepsis. we found that combining PTX3, PCT and lactate, named PPL, significantly improved the performance of these markers along and better than both SOFA and APACHE II scores in predicting in-hospital mortality of adult sepsis.

Despite that the overall mortality rate of septic patients is declining, the incidence of sepsis and the number of sepsis-related deaths are still increasing [15, 16]. It is very essential for risk stratification of sepsis. A review concluded that PTX3 has solid prognostic value in sepsis and correlates with organ dysfunction, but with limited specificity [17]. It has been suggested to be a good predictive marker of mortality in sepsis since its reflection of tissue involvement by inflammatory processes more directly [13, 18]. The most valid cut-off level of PTX3 at admission was 140.28 ng/ml in a recent prospective cohort study [19]. Differently, we identified that PTX3 >49.9 ng/ml was an independent risk factor for 28-day mortality, which was similar with another study [20]. This inconsistence may due to the heterogeneity of different study subjects. We and others [20–22] all showed PTX3 concentrations were quickly reduced during the ICU stay with effective treatment, which may indirectly reflect the severity of sepsis at the onset then to predict the outcome. Moreover, consistent with the other study [23], we also identified PTX3 as an independent risk factor for case fatality after adjusted for confounders. The prognostic value of PTX3 in our cohort was better than that for PCT and lactate during the first days once diagnosed with sepsis. Taken together, these findings confirmed the prognostic value of PTX3 in patients with sepsis/septic shock.

Previous studies had elevated PCT concentrations are associated with all-cause mortality in septic patients [24, 25]. In accordance with these studies, we also found that PCT is a moderate predictor of 28-day mortality (the sensitivity and specificity was 71.1% and 72.7% respectively). In our septic population, the cut-off value of PCT was 3.75 ng/ml. Until now, no consistent optimal cutoff point was identified (from 0.9 to 10 ng/ml) due to the heterogeneity in methodologies [26–29]. Thus, relationship between PCT and prognosis required further studies. In addition, some studies suggested that PCT clearance (reduction in PCT) provides more useful information about 28-day mortality in sepsis [30]. A recent study found that inability to decrease procalcitonin by more than 80% is a significant independent predictor of mortality [31]. Similarly, our study also identified that survivals showed significant higher PCT reduction than non-survivals. Whether PCT clearance possess clinical usage for prognosis prediction and the exact cut-off values needs other prospective studies to validate. The Third International Consensus Definitions for Sepsis and Septic Shock (Sepsis-3) recommends measuring lactate concentrations for septic shock [32]. An elevated blood lactate concentration at any time point must be due to an increase in its production, a decrease in its clearance, or both [33]. Variety of studies had suggested that early changes in blood lactate levels are useful in identifying those at high risk of death [34–36]. Similarly, we demonstrated that initial lactate concentration and lactate clearance were significant univariate predictors of hospital outcome. However, although initial elevated lactate was an independent risk factor for mortality, its predictive value was not sufficient in our study. Conversely, Oh et al. suggested that despite low arterial lactate, patients with a high APACHE II score, had a poorer prognosis [37]. Although lactate levels were intravenously tested in our study. There was no significant difference between venous and arterial measurement of lactate for 30-day mortality [38]. Lactate should be considered as one of the best and easily accessible laboratory markers of sepsis severity and morality [39].

Models that based on the combination of several biomarkers provide a novel and useful strategy to overcome the limited performance of one parameter for a given outcome. In this study, we addressed that multi-marker approach may be an aid for the prognosis prediction and conducted multivariate logistic regression to obtain a new sepsis score PPL (PTX3 + PCT + lactate), which yielded better prognostic value than PTX3, PCT or lactate alone. PPL were even more significant in predicting survival of patients with sepsis than commonly used severity scores (APACHE II or SAPS II). Why are these three biomarkers more specific for sepsis than any other biomarkers? PTX3, PCT are specific for different aspects of the inflammatory process leading to sepsis [40, 41]. With the combination of lactate, which is the reflection of metabolic alterations in patients with sepsis such as increased glycolysis and alterations in pyruvate dehydrogenase activity [42–44], these three would serve to confirm the levels of inflammation and subsequent organ damage, to some degree, but also the nonspecific antibacterial effect.

There are several limitations in the study. The study was conducted in a single center and the patients enrolled had a wide age range. These could be diluted in the larger analysis. In addition, patients were followed up only during stay in hospital. On the other hand, as a part of patients didn't seek medical advice directly in our hospital, the precise interval of time between the onset of infection is difficult to determine. This may translate into underestimate the AUC value of severity scores, study markers and the model. Even so, as available resources vary largely among institutions, each center should define its own cutoff value of predicted mortality to adapt treatment intensity.

In summary, our study confirmed that PTX3 can be considered as a useful biomarker in prediction severity and outcome of patients with sepsis. It is a useful clinical tool for risk stratification. Furthermore, the new developed in-hospital mortality prediction model (PPL) enhanced septic mortality prediction by combining PTX3, PCT and lactate. We believe this prediction model may help in the challenging task of improving the care of critically ill patients with sepsis.

## MATERIALS AND METHODS

### Study population

From April 2016 to February 2017, 245 consecutive patients who diagnosed as sepsis (defined as life-threatening organ dysfunction caused by a dysregulated host response to infection) according to the criteria of Sepsis-3 [32] in the intensive care units (ICU) of West China Hospital of Sichuan University were screened for analysis. Exclusion criteria: (1) younger than 20 years or older than 80 years; (2) moribund patients (death previewed for the next 24 hours); (3) patients who had undergone cardiopulmonary resuscitation within 24 h before ICU admission; (4) patients who were treatment with antibiotics for more than 48 hours before ICU admission; (5) patients with immunodeficiency disorders; (6) incomplete information. Finally, 141 patients were enrolled in the study containing 86 males and 55 females The average age was 64 (range from 33 to 78) years. The primary endpoint of the study was all-cause mortality with a follow up for 28 days. The study procedure was approved by the ethics committee of the West China Hospital of Sichuan University and all subjects in this study signed informed consent.

### Data collection

Clinical data was collected at admission and during the clinical follow-up of patients. Septic shock was identified with persisting hypotension requiring vasopressors to maintain mean arterial pressure ≥65 mm Hg and having a serum lactate level >18mg/dL despite adequate volume resuscitation in septic patients [32]. Sepsis related Organ Failure Assessment score (SOFA)[45] and Acute Physiology and Chronic Health Evaluation II (APACHE II) score [46] were evaluated at the time of inclusion and re-evaluated during the first week. Age, sex, body mass index (BMI), comorbidities at admission, and primary site of infection were recorded by specialists in ICU. Blood samples were serially collected 0, 3 and 7 days after study enrolment (or at ICU discharge, whichever came first), centrifuged (4°C, 3000 r/min, 10 min) and EDTA plasma was shipped on dry ice to a central repository and stored at −80°C until further biochemical analysis. The serum levels of PTX3 was measured with specific sandwich enzyme-linked immunosorbent assays (R&D systems, Minneapolis, USA). A detection limit of 0.1 ng/mL and an inter-plate variance of 8-10% was set, as reported elsewhere. PCT was measured using the electrochemical luminescence method (VIDAS Brahms PCT, Mannheim, Germany) according to the manufacturer's instructions and a detection limit of 0.05 ng/mL was set with an inter-plate variance under 20%. Lactate was tested by a blood-gas analyzer (GEM 3000, USA).

### Statistical analysis

All analyses in this study were conducted using the SPSS Statistics software package V.20 (IBM, Chicago, USA). GraphPad Prism 5.0 (GraphPad Software Inc., CA) were used to plot. Categorical variables are expressed as proportions and were tested using χ2 test or Fisher exact test. Continuous variables are presented as mean ± standard deviation (SD) and the differences in mean were analyzed using Student's t test. Non-normal distributions variables were given as median (range) and compared using Mann Whitney U test. One-way ANOVA or Kruskal-Wallis test were used to compare difference among more than two groups when appropriate. Pearson's correlation analysis was used to analyze the relationship among SOFA score, APACHE II score and PTX3, PCT and lactate. Cox proportional hazards analysis was used to determine the risk factors for 28-day mortality. Receiver operating characteristic (ROC) curves were reported to compare the prognostic value of PTX3, PCT and lactate. Survival analysis was performed using the Kaplan-Meier curve and log-rank test. P values of less than 0.05 was regarded as significant.
